# Bending the curve of biodiversity loss requires a ‘satnav’ for nature

**DOI:** 10.1098/rstb.2023.0210

**Published:** 2025-01-09

**Authors:** Andy Purvis

**Affiliations:** ^1^ Biodiversity Futures Lab, Natural History Museum, London SW7 5BD, UK; ^2^ Georgina Mace Centre for the Living Planet, Silwood Park, Ascot SL5 7PY, UK

**Keywords:** biodiversity models, biodiversity indicators, global biodiversity framework, biodiversity monitoring, IPBES, CBD

## Abstract

Georgina Mace proposed bending the curve of biodiversity loss as a fitting ambition for the Convention on Biological Diversity. The new Global Biodiversity Monitoring Framework (GBMF) may increase the chances of meeting the goals and targets in the Kunming–Montreal Global Biodiversity Framework (KMGBF), which requires bending the curve. To meet the outcome goals of KMGBF, the GBMF should support adaptive policy responses to the state of biodiversity, which in turn requires a ‘satnav’ for nature. The twin pillars of such a satnav are (i) models to predict expected future outcomes of today’s choices; and (ii) rapid feedback from monitoring to enable course corrections and model improvement. These same elements will also empower organizations to ensure that their actions are truly nature-positive, but they are not yet written into the GBMF. Without a satnav, society will effectively have to try to find its way to the outcome goals by looking in the rear-view mirror that the current headline indicators provide. Drawing contrasts and parallels with climate modelling, I discuss challenges for indicators, models, data and research culture that must be overcome if we are to bend the curve, and suggest ways forward.

This article is part of the discussion meeting issue ‘Bending the curve towards nature recovery: building on Georgina Mace's legacy for a biodiverse future’.

## 1. Preamble: Georgina Mace and ‘bending the curve’

Biodiversity and many of the benefits it provides to people are in widespread and rapid decline in the face of multiple intensifying anthropogenic drivers [1,2]. Ecosystem collapse and natural resource shortages are ranked among the five most serious risks to the global socioeconomy over the next decade [3]. We must ‘bend the curve of biodiversity loss’—i.e. reverse the sign of recent global biodiversity trends [4]—if we are to attain the Kunming–Montréal Global Biodiversity Framework’s (KMGBF) vision of a world where ‘by 2050, biodiversity is valued, conserved, restored and wisely used, maintaining ecosystem services, sustaining a healthy planet and delivering benefits essential for all people’ [5].

Although ‘bending the curve’ had previously been used to characterize policy options towards a more sustainable future [6], it was added to the biodiversity lexicon by Georgina Mace [4]. Her work over the preceding three decades was probably as important as anyone’s in demonstrating the need to bend the curve. Given that this article comes from a discussion meeting entitled, ‘Recovering nature: building on Georgina Mace’s work to ensure a biodiverse and liveable future’, I briefly review her contribution to some key milestones towards the KMGBF, while of course recognizing that very many others have also been instrumental.

First, the IUCN Red List categories and criteria, which she designed together with Russell Lande [7], brought objectivity, transparency and generality to the assessment of species' extinction risk. This work drove the first comprehensive assessments of major taxonomic groups [8], which showed alarming numbers of bird and mammal species to be threatened with extinction. It is hard to overstate how influential and important this work has been in global conservation. Furthermore, because the criteria were deliberately so general—based around fundamental (rather than clade-specific) features of life history and ecology—applying them across all animal and plant life is not only possible but valuable [9], and suggests that at least around 1 million animal and plant species are now threatened with extinction [10].

A second major contribution was to develop and support quantitative indicators of biodiversity change. When the CBD agreed the target in 2002 of significantly reducing the rate of biodiversity loss by 2010, few indicators could test whether the target would be met. In direct response [11], Georgina helped to develop the Red List Index, leveraging the objectivity and transparency of the Red List criteria to develop a change indicator from repeated assessments of taxonomic groups [12,13]. She also supported and helped to resource the Living Planet Index (recently reviewed by [14]). These indicators were among the first to put a timescale on biodiversity’s decline.

As a leader in conceptualizing and demonstrating society’s dependency on biodiversity, Georgina also showed why these declines are of more than academic concern. She was a Coordinating Lead Author of the Millennium Ecosystem Assessment [15], which assessed the effects of anthropogenic ecosystem change on human well-being and popularized the concept of ecosystem services. She further developed the thinking around ecosystem services in the conceptual frameworks for two groundbreaking initiatives: the UK National Ecosystem Assessment [16] and the Intergovernmental Science-Policy Platform on Biodiversity and Ecosystem Services (IPBES) [17]. In reformulating the planetary boundary for biodiversity loss, she highlighted how it represents short- and long-term risks to the global socioeconomy [18].

She also advanced the state of biodiversity modelling, recognizing the value to policymakers of biodiversity projections under plausible futures, especially from scenarios with feedbacks between nature and society [19]. She used both highly mechanistic [20] and highly phenomenological [21] models, and inspired the most ambitious global biodiversity model ensemble to date, which identified a set of ambitious actions that may—taken together—be sufficient to bend the curve [22]. This analysis in turn influenced the biodiversity goal of the KMGBF. She also lobbied for the establishment of a global biodiversity observing system able to synthesize flows of new data to track progress towards agreed goals [23]. Georgina’s legacy, then, is not only a more ambitious goal for nature in the KMGBF but also a powerful set of tools and perspectives to help reach that goal.

In this article, I argue that the research and science-policy communities need to go further to enable society to reach the outcome goals of the KMGBF. A step change in biodiversity modelling is required alongside the step change in biodiversity monitoring that the KMGBF already envisages. Such changes are needed to develop what I term a ‘satnav’—a forward-looking but adaptive framework that, like a vehicle’s satellite navigation system, tells us where we are, identifies the actions projected to take us to our desired destination most efficiently, and is sensitive to near-real-time information about our position and possible routes ahead. An analogous combination of modelling and monitoring is used to set and track progress towards climate goals. I focus exclusively on quantitative indicators because modelling has focused on these so far, but the arguments also apply to qualitative indicators that relate predictably to the observed state of nature. I discuss some fundamental reasons why biodiversity-change models are less well developed than climate-change models, along with a range of ways to accelerate their improvement. These include a tighter link with monitoring efforts and a properly funded programme of biodiversity-change model intercomparisons.

## 2. Goals, targets and indicators in the KMGBF

The KMGBF’s goal of bending the curve is more ambitious than any set by previous Convention on Biological Diversity Conferences of the Parties (CBD COPs) [24]. Given this level of ambition, the poor track record of achieving global biodiversity targets is particularly concerning. The target set in 2002 was to significantly reduce the rate of biodiversity loss by 2010; this target was missed [25]. The 20 Aichi Targets agreed in 2010 aimed, by 2020, to reduce indirect and direct drivers of biodiversity loss, halt species extinction, expand the areas under restoration and legal protection and safeguard ecosystems that underpin livelihoods; all 20 targets were missed [26], despite a warning as early as 2014 that progress was not on track [27].

The KMGBF set four goals for the year 2050: goal A relates to biodiversity, goal B to nature’s contributions to people (NCP), goal C to benefit-sharing and goal D to implementation. This article is focused primarily on goal A, which has elements relating to improving the area and condition of the world’s natural ecosystems, reducing species' extinction rates and increasing their population sizes, and maintaining genetic diversity. To increase the prospects of reaching the goals, the KMGBF also set action targets to be completed by 2030 as a pathway to reaching the goals, and announced a new Global Biodiversity Monitoring Framework (GBMF) to track progress [5,28]. A three-tiered framework for quantitative indicators has been established [28]: a minimal set of ‘headline’ indicators that Parties to the CBD should use to track and report progress (table 1 shows those relating to goal A and its targets); a larger set of ‘component’ indicators that cover components of targets and goals and that parties can also optionally track and report; and an even larger set of optional ‘complementary’ indicators for more detailed analysis. By 2025, all indicators in the KMGBF must meet six criteria (table 2; although the caveats in criteria 3 and 6 mean that these two need not in fact be met).

**Table 1.** Headline indicators for KMGBF goal A and its associated targets, for which a current methodology is already agreed [28].

focus	headline indicator(s)
Goal A: integrity, connectivity and resilience of all ecosystems maintained, enhanced or restored, substantially increasing area of natural ecosystem by 2050. Anthropogenic extinction of known threatened species halted by 2050, with the extinction rate and risk of all species reduced by 90% and the abundance of native wild species increased to healthy levels. Genetic diversity maintained within populations of wild and domesticated species.	Red List of Ecosystems
	Extent of natural ecosystems
	Red List Index
	The proportion of populations within species with an effective population size >500
Target 1: plan and manage all areas to reduce biodiversity loss.	Red List of Ecosystems
	Extent of natural ecosystems
Target 2: restore 30% of all degraded ecosystems.	(none)
Target 3: conserve 30% of land, waters and seas.	Coverage of protected areas and other effective area-based conservation measures
Target 4: halt species' extinction, protect genetic diversity and manage human–wildlife conflicts.	Red List Index
	The proportion of populations within species with an effective population size > 500
Target 5: ensure sustainable, safe and legal harvesting and trade of wild species.	Proportion of fish stocks within biologically sustainable levels
Target 6: reduce the introduction of invasive alien species by 50% and minimize their impact.	Rate of invasive alien species establishment
Target 7: reduce pollution to levels that are not harmful to biodiversity.	Index of coastal eutrophication potential
Target 8: minimize the impacts of climate change on biodiversity and build resilience.	(none)

**Table 2.** Criteria that indicators in the KMGBF must meet by 2025 (summarized from [28], with caveats quoted).

Data and metadata publicly available.
Indicator methodology has been peer-reviewed and validated for national use.
Data sources and indicators compiled and updated regularly at least every 5 years ‘if possible’.
The ongoing production of the indicator is guaranteed by an appropriate organization, which provides guidance on its use.
Indicators can detect trends relevant to the KMGBF.
‘When possible’, indicators are aligned with relevant existing intergovernmental processes and related organizations.

In this article, I argue that this minimal set of headline indicators simply cannot provide the timely information on quantitative changes in biodiversity and its drivers that is needed for us to achieve the outcome goal for biodiversity of the KMGBF. In attempting to navigate towards the outcome goal, relying on the headline indicators is like looking only in the rear-view mirror when navigating a car. The next section contrasts two kinds of biodiversity indicators: those that estimate trends by synthesizing time-series data on biodiversity alone, and those that estimate trends as outputs of models explicitly incorporating drivers of change. I argue that only the latter can provide a satnav.

## 3. Compiled and model-based indicators in the KMGBF

All the headline quantitative indicators for the state of biodiversity (i.e. those listed against goal A) are compiled from time-series data on how biodiversity is perceived to have changed. The same was true of all the other biodiversity indicators used to assess whether the 2010 target or the subsequent 2020 (Aichi) targets were met [25,26]. I refer to indicators of this type as ‘compiled indicators’: each indicator is synthesized from time series of matched observations or perceptions of the variable that are the building blocks of the indicator (e.g. successive IUCN Red List assessments for the same species in the case of the Red List Index). I do not mean to imply that compiled indicators simply show changes in the mean observation over time: they often incorporate statistical modelling to (for instance) correct for sampling biases, or to process satellite imagery.

Many of the limitations common to compiled indicators—geographic and taxonomic biases in data, time lags in compilation, synthesis and reporting—can in principle be greatly mitigated through enhanced data collection and aggregation. However, a more fundamental limitation is that compiled indicators are informative only about what has happened so far, not about what might happen in the future or how to change future trajectories. A 2014 assessment of whether the Aichi Targets were likely to be met in 2020 highlighted this limitation [27]: each indicator time series was fitted with a smooth curve, which was carefully extrapolated to 2020. The extrapolations suggested that none of the indicators would reach their targets by 2020 [27]. However, it would not have been possible to assess which courses of action, if any, would have been expected to achieve the target.

The alternative way to estimate a variable of interest is through a model embodying its dependencies on drivers of change [29]; I use the term ‘model-based indicators’ for such estimates. Models vary widely in how they work and how they make predictions: they range from highly mechanistic (i.e. process-based) to purely phenomenological (i.e. pattern-based), and from fully static (modelling net effects on the equilibrium state) to completely dynamic (explicitly considering the time course of all changes) [19,29,30]. Because the indicator is linked by the model to drivers of change, it can be estimated for any time periods for which the relevant driver information is available, even if it lies outside the observational record. Thus, time series of model-based indicators can move seamlessly from the past to the future; all of the indicators projected in [22,31] provide examples, and many more can be found among the component and complementary indicators in the draft GBMF [28]. Multiple possible futures can be compared, meaning that the expected outcomes from alternative candidate policies can be assessed [29,30]. Model-based indicators can look forwards as well as backwards.

This potential was recognized by IPBES, which emphasized the importance of models for a proactive rather than reactive approach to biodiversity conservation [29], and whose list of ‘core’ indicators of drivers, pressures, states, impacts and responses—unlike the GBMF’s ‘headline’ indicators—included a mixture of compiled and model-based indicators [32]. IPBES assessment teams were urged to make use of these core indicators whenever possible, and the first IPBES Global Assessment used both compiled and model-based biodiversity indicators when reporting on trends since 1970 [10]. Later chapters in the assessment took a system perspective to assess plausible futures (exploratory scenarios) and to identify possible routes to sustainability (goal-seeking scenarios), necessarily using model-based indicators.

Achieving the KMGBF also requires being able to look forwards, but all of the headline indicators look backwards. The criteria for inclusion in the GBMF do not include modellability (table 2), even though it is a reasonable criterion [33]; the word ‘model’ does not even appear in the text of the KMGBF. Although there are headline indicators for some of the 2030 action targets to mitigate drivers of biodiversity loss and step up societal responses (table 1), indicators of pressures or response efforts do not provide much information about whether the goals for the state of biodiversity will be met [11].

**Figure 1.**
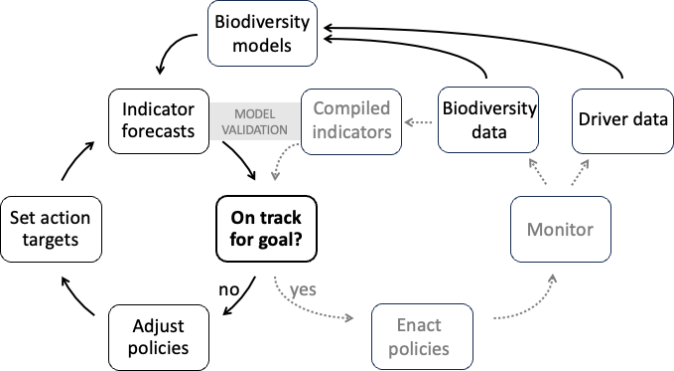
Elements of a satnav for nature that links indicators, goals, targets and policies. The elements shown in black correspond to designing a framework that is expected to meet the specified outcome goals for biodiversity, through iterative formulation of action targets and assessment of their model-projected consequences. Elements in grey show how post-implementation monitoring of both biodiversity and drivers, closely linked to ongoing modelling, allows rapid model improvement, model validation and—more importantly—adaptive policy adjustment to reach the outcome goals, if new indicator forecasts indicate that these will not be met.

Only models can link indicators, goals, targets and policies. As figure 1 shows, they can project whether a set of proposed actions are expected—based on the current understanding of the models embody—to be sufficient to achieve the desired outcome goals. Indeed, through goal-seeking scenario analysis to identify pathways to those goals, they can in principle be used to set action targets that are expected to suffice. This already happens within climate science, where such analysis is the basis of the global stocktake of nationally defined contributions in the Paris Agreement and the reporting framework of the Taskforce for Climate-related Financial Disclosure. The system perspective inherent in models is obviously not sufficient to ensure that the outcome goals will be achieved, but it does enable near-real-time assessments of progress towards them: there is a satnav for the climate.

Why are biodiversity models so far behind? One reason is likely to be funding: the annual expenditure by governments on climate modelling and observation—estimated at around $4 billion—is likely to exceed that devoted to biodiversity modelling [34]. A second, probably related, reason is that biodiversity change is harder to model than climate change. Some of the causes of this difficulty are explored in §4, where we identify steps that would enable and accelerate a satnav for nature.

## 4. Why biodiversity change is harder to model than climate change

The ecological and evolutionary differences among organisms mean that biophysical systems may respond to any given driver of change in a wider range of ways than purely physical systems. This diversity underpins a set of interacting ways in which biodiversity change is harder to model than climate change (table 3). This section explores these and their main consequences for how both modelling and monitoring need to develop to produce a reliable satnav for nature.

**Table 3.** Interacting reasons why biodiversity change is harder to model than climate change.

feature facilitating model development	status in climate change modelling	status in biodiversity change modelling
key variables agreed	yes	partly
key variables scale-independent	yes	no
historical time series available	excellent	poor/absent
geographic uniformity in drivers and responses	approximately	no
anthropogenic drivers commensurable	approximately	no
global indicators agreed	yes	partly
coverage of readily-available new data	excellent	poor
inclusive model intercomparisons	formal process	no formal process

### (a) Agreement on key variables

The climate-change community established a framework of essential climate variables (ECVs) over 20 years ago [35]. This has become a mature and highly influential suite of variables, each making critical, non-redundant and cost-effective contributions to understanding the climate system. As well as variables that capture the state of the climate (such as surface air temperature and precipitation), ECVs also include the concentrations of the greenhouse gases (GHGs) most important in driving anthropogenic climate change: they include both effects and causes. A regular formal process evaluates potential changes to ECVs, so measures that have been made feasible or cheaper by new technologies can be added [35]. ECVs provide a rich basis for comparing, in spatiotemporal detail, many outputs of climate models with observations and with each other.

The success and usefulness of the ECVs inspired the proposal in 2013 of a suite of essential biodiversity variables (EBVs) [36]. As well as starting later, the journey towards a comprehensive set of key variables is harder for biodiversity change than for climate change, both because biodiversity change is more multidimensional and because many of the dimensions are hard to characterize adequately.

The high dimensionality of biodiversity is reflected in the six EBV classes (genetic composition, species populations, species traits, community composition, ecosystem functioning and ecosystem structure) [36]. Each class can be measured in many different ways, and there is often a trade-off between measures readily applied with available data and those that may be more salient from an earth-system or ecosystem-service perspective but whose data requirements restrict their use to the best-studied taxonomic groups and places. As an example, functional or phylogenetic diversity can predict resilient ecosystem functionality better than species richness [37], but requires ancillary information. Although EBVs have proved to be an extremely useful organizing principle for biodiversity measurement, further consolidation is needed.

### (b) Scale-dependency of key variables

In addition to the high dimensionality that the EBV classes reflect, measuring biodiversity is scale-dependent in a way that measuring climate is not: the global total amount of diversity (e.g. number of species) is a quantity of interest that cannot be calculated from a global map of local diversity. This difference has consequences for the choice of essential variables. Whereas local weather stations need to report only a few simple quantities to contribute to a full picture of global climate, local biodiversity cannot be summarized without loss of important information: trends in simple summary statistics (such as measures of taxonomic, functional or phylogenetic diversity) underestimate change because they do not register changes in, for instance, which species are present or numerically dominant [38,39]. Data aggregators therefore need, so far as possible, not to be data simplifierss.

### (c) Historical time series

For any phenomenon being modelled, historical time-series data—especially for long time series—are invaluable for model development, tuning and testing: models that cannot predict what is known to have happened are unlikely to make useful predictions about the future. For climate, weather stations have been recording more or less standard information daily for (in a few places) over 250 years [40]; time series synthesized from these instrumental observations and with good spatiotemporal resolution ([41]) have long been available to modellers. Annual time series have been extended back over thousands of years using proxies such as tree rings [42]; coarser time series from paleoclimate proxies can extend even tens of millions of years in the past. Combining these sources allows inference of global climate back to the Cretaceous–Paleogene boundary [43], enabling models to be tested well outside the time range considered in their development [44].

Nothing even remotely comparable exists for biodiversity. Few current systematic monitoring schemes (which are usually taxon-specific anyway) go back more than 25 years. Although there are occasional exceptions [45], most time series are far shorter—and may be less consistent in terms of methodology—than those available for climate [46]. Fundamentally, very few biodiversity observations were collected as time-series data in the way that weather station records were. Not only is there no temporally well-resolved observational or proxy time series of how biodiversity (rather than a particular taxonomic or ecological group) has changed quantitatively—globally, in any region or arguably even in any locality—since 1900 C.E. or 1000 C.E., but there never will be. Monitoring from now on will have the be the main source of adequate time series.

### (d) Geographic variation in drivers and responses

The main direct anthropogenic drivers of climate change are emissions of GHGs [47]. Although GHG emissions arise from particular places, atmospheric mixing tends to homogenize their concentrations (e.g. mid-tropospheric mean *p*CO_2_ from 2003 to 2011 varied globally by a little over 1% [48]). Atmospheric and oceanic circulation also move heat from low to high latitudes, helping to make the climate a truly global system. This means that climatic responses to drivers of change, as well as the main anthropogenic drivers themselves, are largely (though not exclusively) global.

By contrast, the main direct anthropogenic drivers of biodiversity loss worldwide—land/sea-use change, direct exploitation of organisms, pollution, climate change and invasive alien species [49]—all show strong geographic variation in their intensity [50]. They also have inherently different spatial grain, with the first two being more localized, climate change being much less so and pollution ranging from the highly localized effects of a point-source release to the very widespread effects of atmospheric nitrogen deposition. The differences in spatial grain mean that the main-effect impacts of the different drivers are most easily estimated from very differently designed datasets. Case-control comparisons suit fine-grained drivers well but are much less suitable for coarse-grained drivers, as affected and unaffected sites would have to be far apart [51]. Such discrepancies mean that no current biodiversity model frameworks treat the main direct drivers of biodiversity loss on a level playing field: the structure of the data gives more power and precision to some drivers than to others. For example, analysis of the PREDICTS database [52] showed a significant interaction between land-use change and climate change in reshaping insect assemblages [53]; while such an analysis can estimate the main effect of land-use change and its interaction with climate change, it can neither detect nor estimate the main effect of climate change (because sites sampled within any given study will all have experienced very similar degrees of climate change [51]). Models that instead estimate the effect of each driver from a different model based on different data having the required characteristics (as GLOBIO does, for example [54], by fitting separate models to estimate the impacts of land use, roads, habitat fragmentation, hunting, atmospheric nitrogen deposition and climate change) will struggle to estimate interactions and run the risk of overestimating impacts of strongly correlated drivers.

Biotic responses to these direct drivers will also vary widely from place to place because of geographic variation in other interacting drivers, in the starting level of biodiversity and in its intrinsic susceptibility [55]. Teleconnections linking terrestrial and marine realms and the biomes they contain are weaker effects than in the climate system: biotic responses to these drivers are therefore largely local or at the landscape/seascape scale rather than being global [56]. As a consequence, knowing the trends in biodiversity and its direct drivers within a single region of the earth is much less informative of global trends than would be the case with climate.

### (e) Commensurability of anthropogenic drivers

Carbon dioxide is not the only GHG whose atmospheric concentration is being increased by human actions. The Kyoto Protocol also includes the five next most important GHGs (CH_4_, N_2_O, hydrofluorocarbons, perfluorinated compounds and SF_6_) [57], which differ in both their residence time in the atmosphere and their ability to trap infra-red radiation. However, their expected impacts can be expressed in a common currency such as their global warming potential, i.e. how much energy a unit quantity of gas will absorb over a given time [58]. These currencies are not perfect: debate continues about how best to capture complexities such as interactions and variation in residence times among GHGs and their sources [59,60]. However, even an approximately consistent currency greatly simplifies both the aggregation of GHG emissions from different sectors, companies and countries into global totals, and the disaggregation of global targets to national contributions [61].

The main direct drivers of biodiversity loss—land/sea-use change, direct exploitation of organisms, climate change and pollution—are much harder to convert into an analogous ‘biodiversity loss potential’. Whereas GHGs act through much the same physical pathway and have much the same effect wherever they come from, these drivers act through a wide range of ecological pathways, and their location is critical to their impact. Non-additive effects of multiple drivers add further complexity [62].

### (f) Agreement on global indicators

Through six reporting cycles, the Intergovernmental Panel on Climate Change (IPCC) has developed and refined a set of global indicators of climate change and its main anthropogenic drivers that are estimated by synthesizing observations and model outputs [47] and updated annually [63].

Biodiversity indicators have developed in the absence of rich historical data so, rather than being a distillation of the best quantities for characterizing the system, they have been strongly shaped by the availability of data originally collected for other purposes [23,64]. Although the headline indicators for the KMGBF are intended to enable assessment of progress towards the goals and targets, not all are well aligned with their corresponding target or goal [65].

The planetary boundaries framework represents an attempt to select global indicators based on their importance in governing the behaviour of the Earth system [66]. It proposed using control variables argued to govern the responses of nine Earth systems as they approach destabilizing thresholds. The control variables proposed for biotic integrity were the global rate of species extinction and the global average Biodiversity Intactness Index (BII, a measure of ecological integrity [66,67]). However, neither is obviously a control variable for any part of the Earth system *per se* [56], although the BII may be a useful measure of ecosystems’ capacity to contribute to NCP in the short to medium term [18]. The framework explicitly recognizes that biotic integrity is jeopardized by the transgression of any of the other boundaries [66], highlighting the range of drivers that—because of their impact on biodiversity trajectories—must also be quantified.

### (g) Coverage of readily available new data

Biodiversity science also trails climate science by decades in monitoring [34,68]. One result is that much of the effort needed to produce many biodiversity indicators—whether compiled or model-based—is spent finding and harmonizing relevant biodiversity observations that have already been made. ‘Big data’ pipelines such as eBird [69] show how rapidly biodiversity observations can be aggregated in near real time across much of the globe, but still do little to lessen taxonomic and geographic biases in coverage. Such biases undermine trend estimates from compiled indicators [70]. Spatiotemporally resolved data are also lacking for many drivers of biodiversity change [50], undermining model-based indicators. Systematic monitoring of biodiversity *and drivers*, generating streams of harmonized essential variables in standardized formats, will transform the accuracy, precision and timeliness of compiled and model-based indicators alike [23,68,71], especially if some of the monitoring focuses on minimizing uncertainty [72]. Importantly, indicator estimates compiled from monitoring data can be used to validate model-based estimates (figure 1), highlighting model deficiencies. In this context, the limited teleconnection of global biodiversity is beneficial rather than a handicap: monitoring in many different places will quickly compensate for the shortage of historical data, enabling more precise attribution of changes to drivers and strengthening models [68]. GEO BON’s ‘BON in a Box’ initiative (https://boninabox.geobon.org/) aims to provide an architecture and open tools for integrating accelerating flows of data into EBVs and indicators.

### (h) Maturity of model intercomparisons

IPCC reports first used projections from a model ensemble in 1992 [73]. The Coupled Model Intercomparison Program (CMIP) began in 1996, building on a global collaboration that had compared over 30 atmospheric General Circulation Models based on their outputs given a standard set of inputs (reviewed by Touzé-Peiffer *et al*. [74]). CMIP has greatly helped modellers to understand and improve model performance. Models that do not continue to develop risk being downweighted in ensemble projections; continued relevance thereby provides a strong incentive for ongoing improvement of how models estimate indicators.

Biodiversity model intercomparisons lag behind those for climate. The added difficulty (relative to climate) of modelling biodiversity means that models are not set up well to run common experiments with shared input data and output variables [19,22,30,72]: they project different indicators, having considered different sets of drivers and processes. Outside of species distribution modelling [75], there have been relatively few published formal model comparisons, and projections from ensembles of global biodiversity models were first used in IPBES reports only in 2019 [1].

## 5. Prospects

It is often said that we can only manage what we can measure. However, it would be more accurate to say that we can only manage what we can both measure *and model*. By itself, measurement can tell us that something needs to be done; but deciding what that something is requires an additional ability to project future consequences of alternative choices [76]. A true satnav for nature will further require ongoing monitoring to check that the choice made has the consequences that were expected. While having a satnav is not enough to guarantee reaching the desired destination (as slow progress on climate mitigation makes all too clear), not having one makes it impossible to get there efficiently.

Given the difficulties of modelling biodiversity discussed above, the necessary changes will obviously require more investment and more recruitment [30,68,72]—to build, support and sustain a strong open community of practice. They will also need a balance of support, rights and responsibilities that is fair to generators of primary data, modellers and infrastructure providers. But even ahead of those, some decisions to be taken in the near future could speed up progress by signalling the direction of travel. Guidance on how to implement the GBMF is still being developed, and could make clear the importance and value of model-based indicators in assessing progress towards the KMGBF’s goals for nature, effectively broadening monitoring to include model-based estimation. Likewise, the second IPBES Global Assessment could focus more on the connections between drivers, nature and NCP than did its predecessor, which treated them largely in isolation.

In the medium term, biodiversity modellers should engineer their models to make use of new scenario frameworks that put nature at the centre of the socioeconomy [77], and to estimate one or more of the headline indicators in addition to their measures of choice [33]. A torrent of new monitoring data on biodiversity and drivers, efficiently made available for synthesis and modelling, would rapidly mitigate the absence of historical data. A well-resourced programme of model development and intercomparison would further accelerate model improvement.

Bending the curve of biodiversity loss, then, will require transformational change—not just in the relationships between global society and nature [1,2], but also within the field of biodiversity science. We can—again—take inspiration from Georgina Mace, who always developed her science in the direction of greater relevance to global challenges, irrespective of whether doing so was within her own comfort zone.

## Data Availability

This article has no additional data.
